# Energy balance in women during polar trekking—The POWER study

**DOI:** 10.14814/phy2.70443

**Published:** 2025-06-30

**Authors:** Pierre Bourdier, Jessica Devitt, Susan Gallon, Alexandre Zahariev, Isabelle Chery, Jacob R. Guzzetti, Stéphane Blanc, Chantal Simon, Audrey Bergouignan

**Affiliations:** ^1^ Université de Strasbourg, CNRS, IPHC UMR7178 Strasbourg France; ^2^ Université de Haute‐Alsace, IRIMAS UR7499 Mulhouse France; ^3^ Department of Family Medicine University of Colorado, Anschutz Medical Campus Aurora Colorado USA; ^4^ Network of Marine Protected Areas in the Mediterranean (MedPAN) Marseille France; ^5^ Behaviour‐Brain‐Body Research Centre University of South Australia Adelaide Australia; ^6^ CarMen Laboratory, INSERM 1060, INRAE 1397 University of Lyon Oullins France; ^7^ Human Nutrition Research Centre of Rhône‐Alpes Hospices Civils de Lyon Lyon France; ^8^ Division of Endocrinology, Metabolism and Diabetes Anschutz Health & Wellness Center, University of Colorado, Anschutz Medical Campus Aurora Colorado USA

**Keywords:** body composition, energy balance, physical activity, polar environment, women

## Abstract

Polar expeditions pose a significant challenge, contributing to a substantial energy deficit. However, few data exist on nonprofessional individuals, and none of them have investigated the regulation of energy balance in female participants during an Arctic expedition. Eleven nonathlete female adults who reached the North Pole by ski in full autonomy were studied. Before and the day after the expedition, resting metabolic rate (RMR) was measured by indirect calorimetry, and fat mass (FM) and fat‐free mass (FFM) by bioelectric impedance. Total daily energy expenditure (TDEE) and activity‐related energy expenditure (AEE) were assessed with the doubly labeled water (DLW) during the expedition. Before and throughout the expedition, daily physical activity was evaluated using accelerometry and heart rate, while surface skin temperature was measured using an iButton placed on the chest. Additionally, fasting salivary cortisol concentration was measured throughout the expedition as a stress marker. The seven‐day trekking was associated with a TDEE of 18.67 [SD 1.72] MJ/d. Body mass decreased by 1.67 (SE 0.42) kg, mostly due to fat mass loss (−1.26 [0.39] kg), indicating a negative energy balance. These findings show that nonathlete females can greatly increase their AEE and, hence TDEE over a short time period. However, their food consumption was insufficient to meet their needs, thus leading to an energy deficit and body mass loss. Future studies should investigate whether this insufficient energy intake was due to a lack of available food or an inability to consume more, thereby deepening our understanding of energy balance regulation in extreme conditions.

## Graphical abstract

Polar trekking is characterized by a negative energy balance. While it can be sustained during a few days, prolonged periods of energy deficiency can lead to adverse health effects.

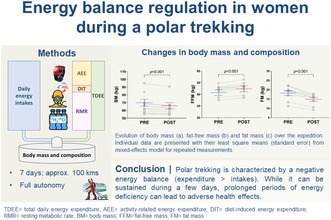

## 1 INTRODUCTION

Polar regions are among the most extreme environments on Earth due to their unforgiving topology, harsh climate, and unique challenges (Cotter & Tipton, 2014). During the “heroic age” of polar exploration (1895–1922) (Leon et al., 2011), many expeditions sought to conquer the Poles and set records, often at great physical cost. Emaciation was a common issue, with severe consequences ranging from compromised health to death (Halsey et al., 2015), particularly when body mass loss reached critical levels. Such loss arises from an energy imbalance, where energy intakes (EI) fail to meet total daily energy expenditures (TDEE), either due to excessive energy demands and/or insufficient food availability.

The Arctic presents a unique array of hazards, including extreme temperatures, blizzards, and unstable terrain marked by crevasses, frozen lakes, and channels of open water. Individuals unaccustomed to such conditions face heightened vulnerability to heat loss, which can impair metabolic, cardiovascular, respiratory, and immune functions (Færevik et al., 2013; Frank, 2001; Leon et al., 2011). To survive, thrive, and perform in these environments, the human body must acclimate (Halsey & Stroud, 2012) by activating physiological compensatory mechanisms such as vasoconstriction, shivering, and brown adipose tissue activation (Leppäluoto & Hassi, 1991; Yurkevicius et al., 2022). Collectively, these mechanisms aim to maintain core temperature at thermoneutrality and are at the origin of cold‐induced thermogenesis. This thermogenesis significantly increases energy expenditure (through resting energy expenditure and non‐exercise activity thermogenesis) (Haman & Blondin, 2017; Kulterer et al., 2020; Ouellet et al., 2012), contributing to higher overall energy requirements. Additionally, the use of high‐performance polar suits, while necessary for insulation, can reduce locomotor efficiency, increasing workload and activity‐related energy expenditure (AEE) by as much as 16% (Holmer, 2009; Teitlebaum & Goldman, 1972). Stressors such as environmental dangers and disrupted sleep patterns (e.g., polar bear watches to prevent attacks, long daylight) may further compound energy demands, as stress and sleep alterations are known to elevate energy expenditure (Chaput et al., 2023; Rabasa & Dickson, 2016).

The need for self‐sufficiency further constrains Arctic expeditions. Remote access typically necessitates foot travel or cross‐country skiing, with explorers hauling sleds containing food, clothing, and tents. This logistical challenge forces participants to optimize their load by balancing the weight of food rations against their energy density (Ahmed et al., 2020; Charlot, 2021). However, inadequate nutrition has historically undermined the physical and psychological resilience of expedition members, jeopardizing mission success and, in extreme cases, leading to fatal outcomes (Halsey & Stroud, 2012). Accurate measurement of energy requirements is, therefore, critical to ensure the safety and performance of polar explorers.

Despite the importance of understanding energy balance in such settings, research in polar environments remains limited. Most existing studies have focused on Antarctica (which is harsher than the Arctic because of extreme weather—up to −60°C with moderate‐to‐strong winds and dry air, isolation—no permanent population, and topology—ice covered land with high summits), involved small sample sizes, and predominantly examined male military or athletic populations (Coker et al., 2025; Hattersley, Wilson, Thake, et al., 2019; Iuliano & Ayton, 2015; Stroud, 1998). Research on women, especially nonathlete or nonmilitary participants, is exceedingly rare (Hattersley et al., 2020; Hattersley, Wilson, Gifford, et al., 2019; Paulin et al., 2015), leaving a significant knowledge gap.

To address this gap, we conducted the POWER study, which followed an all‐female team of 12 European and Middle Eastern explorers during their North Pole expedition in April 2018. This unique expedition, designed to foster dialogue between the East and West, offered an exceptional opportunity to study the physiological adaptations of women to the Arctic environment. The POWER study aimed to determine TDEE and its components (i.e., AEE and resting metabolic rate—RMR), along with changes in body composition, to assess energy balance during the expedition. Other behavioral and biological factors known to influence TDEE, such as daily physical activity, stress, and thermoregulation, were evaluated.

This study represents a critical step toward understanding the energy balance regulation of women in extreme environments, ensuring their safety and success in polar expeditions.

## 2 MATERIALS AND METHODS

### 2.1 Participants

Twelve nonathlete women (i.e., not specifically trained to achieve a high volume of physical activity in extreme environments) participating in the Women's Euro‐Arabian North Pole Expedition 2018 voluntarily took part in the present study. No inclusion or exclusion criteria were established since the POWER study was integrated into the already planned expedition. Participation in the POWER study (for Physiological adaptatiOns in WomEn during a NoRth Pole expedition) was entirely voluntary; participants were free to accept or decline participation and could withdraw at any time without affecting their involvement in the expedition. Baseline analyses were collected on 11 participants. One participant had to be evacuated by helicopter after 2 days of the expedition due to frostbite.

The study was approved by the Colorado Multiple Institutional Review Board (COMIRB) under protocol number 17‐2144. Written informed consent was obtained from all participants in accordance with the principles of the Declaration of Helsinki.

### 2.2 Protocol

Between 14 and 5 days prior to the start of the expedition, participants met in Longyearbyen, Svalbard, Norway, with the scientific team for a briefing about the study and the expedition and to perform baseline data measurements. On April 14, 2018, participants flew on a cargo airplane to Camp Barneo, a private temporary tourist resort located on Arctic Ocean ice near the North Pole. The expedition aimed to reach the geographic North Pole with full autonomy, with participants skiing 6–7 h per day and pulling their 70 kg sledges containing their own food, clothing, and ice tents for the entire journey. Over 7 days, the team covered a distance of 60 nautical miles (approximately 100 km) and reached the North Pole on April 22, 2018.

Data collection was conducted at the Longyearbyen Hospital both before the expedition and the day after the participants returned to Longyearbyen, that is, 1 day after they reached the North Pole. Body mass, body composition, and RMR were measured before and after the expedition. Free‐living TDEE during the expedition was measured with the doubly labeled water (DLW) method. Upon their initial arrival in Longyearbyen, participants were equipped with loggers to continuously measure daily physical activity, heart rate, and skin surface temperature. The day before their departure to Camp Barneo, participants were given new monitors and instructed to wear them throughout the expedition.

### 2.3 Total energy expenditure

TDEE during the expedition was determined using the DLW method over 8 days, encompassing the day before the expedition and the 7 days of the expedition. On the morning prior to the expedition, participants ingested a premixed 2.68 g/kg estimated total body water (TBW) composed of 0.33 g/kg TBW H_2_^18^O enriched at 13.4% and 0.22 g/kg TBW of ^2^H_2_O enriched at 99.9% (Eurisotop, Saint‐Aubin, France), ensuring sufficient enrichment during high water turnover rates. Saliva samples were collected with salivettes 4 and 5 h after dose ingestion to calculate equilibration (Blanc et al., 2002), and subsequently on day 9 and day 9 + 1 hour post‐dose.

Saliva samples were centrifuged and filtered to extract water. Hydrogen and oxygen isotope ratios were measured using a high‐temperature conversion elemental analyzer (TC/EA) interfaced with a Delta V Plus Isotope‐Ratio Mass Spectrometer and a Conflo III interface (THERMO, Bremen, Germany). Results were scaled using two internal laboratory standards normalized against international standards versus the VSMOW2/SLAP2 scale (International Atomic Energy Agency, Vienna, Austria). All analyses were performed in quadruplicate and repeated if the standard deviation (SD) exceeded 2% for deuterium and 0.2% for 18‐oxygen.

TDEE was calculated using the multipoint DLW approach. Dilution spaces for ^2^H and ^18^O were derived from the baseline and equilibration samples, according to Coward et al. (1994). TBW was calculated from the average of deuterium and 18‐oxygen dilution spaces after correction for isotope exchange by 1.041 and 1.007, respectively (Bhutani et al., 2015; Racette et al., 1994). Constant elimination rates for ^2^H and ^18^O were calculated by least‐squares linear regression on the natural logarithm of isotope enrichments from all urine samples as a function of elapsed time from dose administration. CO_2_ production was then estimated according to Schoeller (1988) modified by Racette et al. (1994), and TDEE was derived from Weir's equation (Weir, 1949) using a food quotient (FQ). Macronutrient composition was extracted from each prepackaged meal to calculate the FQ of each meal using Black's formula (Black et al., 1986) (Table S1), and the average FQ was used for TDEE calculation.

Due to potential changes in the isotopic enrichment of environmental water during the expedition (Stroud et al., 1993), ice samples were collected daily to correct for background isotopic enrichment. Additionally, a control participant who did not receive the DLW dose followed the same urine collection schedule as the subjects who received the dose. This provided corrections for changes in background isotopic enrichment, resulting in a sample of 10 participants who were dosed.

### 2.4 Resting metabolic rate and activity‐related energy expenditure

RMR was measured before and after the expedition using indirect calorimetry (Fitmate, COSMED, Rome, Italy). Participants were instructed to lie down, remain awake, and avoid speaking during the measurement, which was conducted under thermoneutral conditions. The inspired fraction of oxygen was recorded over a 25‐min period, with the first 5 min excluded from the analyses to ensure steady‐state measurement. Oxygen consumption (VO_2_) was calculated using Lighton's equations (Lighton, 2008), while carbon dioxide production (VCO_2_) was derived from VO_2_ using a respiratory quotient estimated from the FQ. Energy expenditure was then calculated using Pérronnet & Massicotte's equation (Péronnet & Massicotte, 1991).

Physical activity level (PAL) was calculated as the ratio of TDEE to RMR. Non‐resting energy expenditure (TDEE‐RMR) corresponds to the energy cost associated with exercise activity and thermogenesis linked to non‐exercise activity thermogenesis, thermoregulation in response to the polar environment, and the cost of food processing (digestion, assimilation, utilization, and storage). Diet‐induced thermogenesis is assumed to be on average 10% of TDEE, which allows for estimating AEE as 0.9 × TDEE‐RMR. Notably, AEE represents not only the energy budget allocated to exercise and non‐exercise physical activities but also the cost of thermoregulation.

### 2.5 Energy intake and energy balance

Prepackaged food had an average composition of 28% fat, 55.8% carbohydrates, and 16.2% protein. The proportion of macronutrients and the number of calories for each prepacked meals are detailed in Table S1. Participants carried approximately 2.6 kg of food rations for the trekking, for a total energy of 42 MJ, resulting in an average FQ of 0.89. Since food records during the expedition were not sufficiently accurate, energy intake (EI) was estimated based on changes in body composition and TDEE between the baseline and post‐expedition measurement sessions (Ravelli & Schoeller, 2021). Energy balance (EB) was calculated as the difference between EI and TDEE.

### 2.6 Type and intensity of physical activity

Daily physical activity was evaluated using tri‐axial accelerometers (ActiGraph GT3x+, ActiGraph®, Pensacola, FL, USA) worn on the waist during the baseline and throughout the expedition. The companion ActiLife software V.6.4.3 was used to obtain the daily vector magnitude in counts per minute (VM, cpm) as a proxy for overall physical activity. Light‐, moderate‐, and vigorous‐accelerations‐related physical activities were categorized using cutoff values as described previously (De Jong et al., 2018). Because participants had to carry heavy loads over challenging terrain, their walking pace was relatively slow, resulting in light/low accelerations. However, these low accelerations do not correspond to light‐intensity physical activities, as typically defined. Therefore, we categorized physical activities based on light, moderate, and vigorous accelerations rather than referring to “intensity”, which traditionally refers to an estimated volume of oxygen consumption in studies with accelerometers. Heart rate was continuously monitored during the same timeframe using a chest‐worn heart rate sensor (Polar H7, Polar Electro, Kempele, Finland). Median heart rate per hour was calculated and matched with the corresponding hourly accelerometer counts as a proxy of physical activity intensity.

### 2.7 Body mass and composition

Participants were weighed before and after the expedition in undergarments, following an overnight fast, using a standard scale. Fat‐free mass (FFM) and fat mass (FM) were measured using bioelectric impedance analysis (BC‐601 Body Composition Monitor, Tanita, Tokyo, Japan). Body composition accuracy may be impacted in case of dehydration of the volunteers upon their return. However, the protocol was run in Longyearbyen, which provided easy access to water on the days preceding and following the expedition, which provided participants sufficient time to drink enough water to rapidly recover from any potential partial dehydration; studies showed that mild hydration can be recovered by oral water intake within few hours (Fan et al., 2020).

### 2.8 Skin temperature

Skin temperature was continuously recorded before and during the expedition with loggers (iButton Temperature, DS1922L, Sunnyvale, CA, USA) strapped to the chest, with an accuracy of +/−0.5°C. Two iButtons were also attached to two different sledges to measure ambient temperatures.

### 2.9 Salivary cortisol

Starting the day before the expedition and continuing every morning during and the day after the expedition, participants were asked to collect duplicate saliva samples using two salivette tubes. Samples were taken immediately upon waking and 30 min later while participants remained in bed or sleeping bags without standing up. Saliva cortisol concentrations were measured with an ELISA kit (catalog# 1–3002, Salimetrics, Carlsbad, CA, USA). Intra‐ and inter‐assay precision ranged from 3% to 7% and 3% to 11%, respectively, with an analytical and functional sensitivity of 0.007 μg/dL and 0.028 μg/dL, respectively. The mean value of the two concentrations was used for analyses.

### 2.10 Sleep

Participants were asked to wear wrist‐actigraphy (Actiwatch Spectrum Plus, Philips Respironics, Inc.; Murrysville, PA, USA) to assess baseline sleep for ~1 week in Longyearbyen and during the expedition from Camp Barneo to the North Pole in constant daylight. Data were collected continuously at 1‐min intervals throughout the study period.

Using the medium sensitivity threshold (Philips Actiware 6, version 6.0.4), sleep periods were manually selected based on 10 consecutive 1‐min epochs of 0 activity counts, indicating the start and end of each sleep episode. The sleep duration of naps was appended to the sleep duration the night prior.

Sleep regularity was assessed using the Sleep Regularity Index (SRI) (Phillips et al., 2017). The SRI makes no structural assumptions of sleep and calculates the chance of being awake or asleep at any two‐time points 24 h apart (Phillips et al., 2017). Data loss precluded the assessment of SRI for three participants during baseline data collection and one participant during the expedition. The first and last days of actigraphic data were excluded from the SRI calculation, as devices were provided/collected on these days, thus not all time asleep may have been captured.

### 2.11 Statistical analyses

Visual inspection of the histogram of residuals and analysis of Kurtosis and Skewness indexes, as Shapiro–Wilk tests, were used to assess the normality of data distribution. Linear mixed‐models (LMM) with repeated measurements (baseline vs. post‐expedition) and time as a fixed effect were used to test the effect of the polar expedition on the variables. A compound‐symmetric covariance structure was used to account for dependencies among repeated measurements. GLMs were also used to examine the association between heart rate and accelerometer counts before and during the expedition. To compare the slopes of these associations across periods (i.e., baseline vs. expedition), ANCOVA was performed, including an interaction term (heart rate * period interaction).

Baseline values are presented as means (standard deviation, SD). Unless otherwise specified, other data are presented as least‐squares means (standard error) (LS means [SE]) or with their 95% confidence interval (CI). Statistical inferences were made on changes induced by the expedition (i.e., delta between pre and post‐expedition). Analyses were performed using SAS version 9.4 (SAS Institute, Cary, North Carolina, USA) and R Studio version 2024.12.0 + 467 for Windows (Posit Software, PBC, Boston, MA, USA), with a significance level of 5%. Figures were generated using Prism 10.0.0 (GraphPad, San Diego, CA, USA).

## 3 RESULTS

### 3.1 Baseline characteristics of participants

Eleven women aged 36.9 years (SD 6.7; range: 28–49 years) completed the study. Participants were normal‐weight, with a mean body mass index of 24.2 (3.4) kg/m^2^ (range: 21.6–30.7 kg/m^2^). They had a fat‐free mass index of 15.8 (1.0) kg/m^2^ (range: 14.5–17.5 kg/m^2^) and a fat mass index of 7.5 (2.7) kg/m^2^ (range: 5.2–13.2 kg/m^2^). Mean adiposity was 30.4 (6.1) % (range: 23.8%–43.5%) (Table 1). Before the expedition, while in Longyearbyen, participants spent an average of 14.6 (3.4) h/d in sedentary activities. Time spent in light‐, moderate‐, and vigorous‐acceleration derived physical activities was 1.30 (0.23) h/d, 1.42 (0.16) h/d, and 0.11 (0.08) h/d, respectively. Participants averaged 6.49 h of sleep per night (SD = 0.56; range 5.77–7.40) with an SRI score of 85.71 (SD = 3.57; range 80.90–89.38), indicating good sleep regularity.

**TABLE 1:** Baseline characteristics of the participants (values are presented as mean [standard deviation]).

	*n*	Mean (standard deviation)	Minimum—Maximum
Demographic outcomes			
Age (year)	11	37 (7)	28–49
Height (m)	11	1.66 (0.07)	1.57–1.77
Anthropometric outcomes			
Body mass (kg)	11	66.63 (12.33)	55.00–94.80
Fat‐free mass (kg)	11	43.45 (4.38)	35.70–50‐80
Fat mass (kg)	11	20.85 (8.56)	13.30–41.24
Fat mass (%)	11	30.41 (6.14)	23.80–43.50
Body mass index (kg/m^2^)	11	24.21 (3.40)	21.59–30.71
Fat‐free mass index (kg/m^2^)	11	15.84 (1.04)	14.83–17.48
Fat mass index (kg/m^2^)	11	7.53 (2.69)	5.18–13.16
Energetic outcomes			
Estimated total energy expenditure (MJ/d)^a^	10	9.15 (1.51)	6.59–12.01
Estimated activity‐related energy expenditure (MJ/d)^a^	10	2.13 (0.35)	1.54–2.80
Resting metabolic rate (MJ/d)	10	6.16 (1.00)	4.34–8.09
Accelerometer‐related physical activities			
Light accelerations‐related activities (h/d)	9	1.29 (0.23)	0.91–1.58
Moderate accelerations‐related activities (h/d)	9	1.42 (0.16)	1.14–1.67
Vigorous accelerations‐related activities (min/d)	9	6.42 (4.99)	2.17–17.08
Sedentary behavior (% of daily activities)	9	82.72 (4.35)	74.20–88.51
Sleep outcomes			
Sleep duration (hour/d)	10	6.49 (0.56)	5.77–7.40
SRI score	7	85.71 (3.57)	80.90–89.38

^a^ Estimated as RMR *PAL (PAL = 1.5).

### 3.2 Expedition‐induced changes in body mass and composition

**FIGURE 1:**
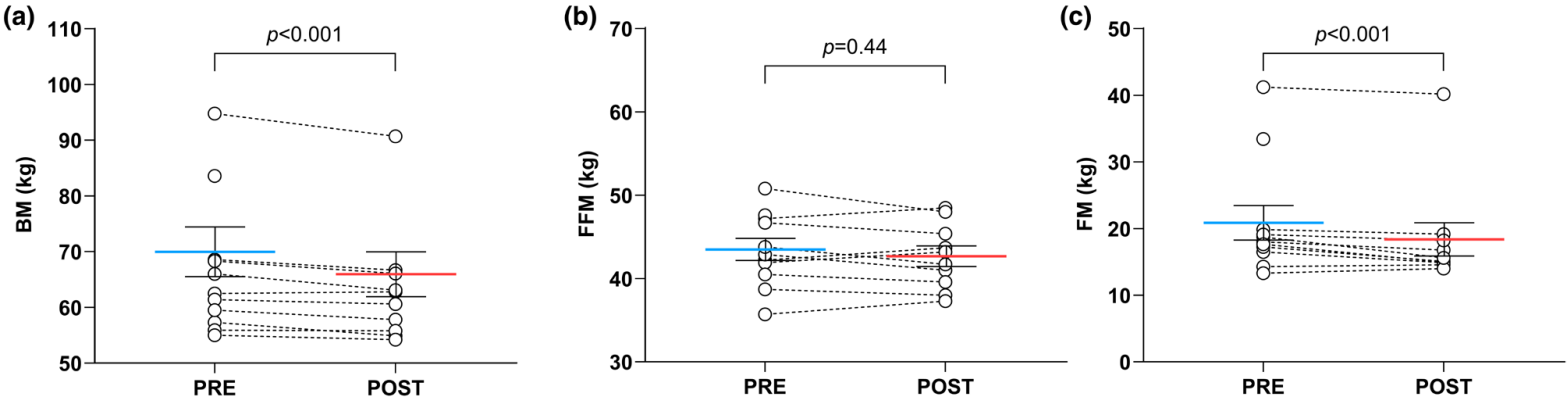
Evolution of body mass (a), fat‐free mass (b), and fat mass (c) over the expedition. Individual data are presented with their least square means (standard error) from mixed‐effects model for repeated measurements. BM, body mass; FFM, fat‐free mass; FM, fat mass.

During the expedition, body mass decreased by 1.67 (SE 0.42) kg (95% CI −2.63 to −0.71), primarily due to a loss of FM (−1.26 [0.38] kg; 95% CI −2.13 to −0.39). No significant change was observed in FFM (−0.43 [0.53] kg; 95% CI −1.64 to 0.77) (Figure 1, Table 2).

**TABLE 2:** Changes induced by the expedition (values are presented as LS means [standard error]).

	*n*	LS means during the expedition (standard error)	Estimated change from baseline (standard error)	95% confidence interval	*p* Value
Anthropometric outcomes					
Body mass (kg)	11	64.96 (3.60)	−1.67 (0.42)	−2.63 to −0.71	0.003
Fat‐free mass (kg)	11	43.02 (1.26)	−0.43 (0.53)	−1.64 to 0.77	0.435
Fat mass (kg)	11	19.59 (2.57)	−1.26 (0.38)	−2.13 to −0.39	0.010
Fat mass (%)	11	29.13 (1.90)	−1.28 (0.69)	−2.83 to 0.28	0.100
Energetic outcomes					
Total energy expenditure (MJ/d)	9	18.92 (0.54)			
Activity‐related energy expenditure (MJ/d)	9	10.78 (0.32)			
Resting metabolic rate (MJ/d)	10	6.31 (0.29)	0.15 (0.19)	−0.28 to 0.59	0.277
Estimated energy intakes (MJ/d)	10	13.00 (1.86)			
Water turnover (L/d)	8	2.95 (0.26)			
Accelerometer‐related physical activities					
Light accelerations‐related activities (h/d)	10	6.13 (0.22)	4.82 (0.30)	4.15 to 5.49	<0.001
Moderate accelerations‐related activities (h/d)	10	2.14 (0.23)	0.73 (0.32)	0.01 to 1.45	0.047
Vigorous accelerations‐related activities (h/d)	10	0.02 (0.02)	−0.08 (0.02)	−0.14 to −0.03	0.008
Sedentary behavior (% of daily activities)	10	59.52 (1.29)	−23.28 (1.98)	−27.73 to −18.87	<0.001
Sleep outcomes					
Sleep duration (hour/d)	10	6.99 (0.20)	0.50 (0.15)	−0.17 to −0.82	0.008
SRI score	9	65.97 (2.38)	−17.95 (2.85)	−25.82 to −10.08	0.003

### 3.3 Daily physical activity, energy expenditures, and energy intake during the expedition

**FIGURE 2:**
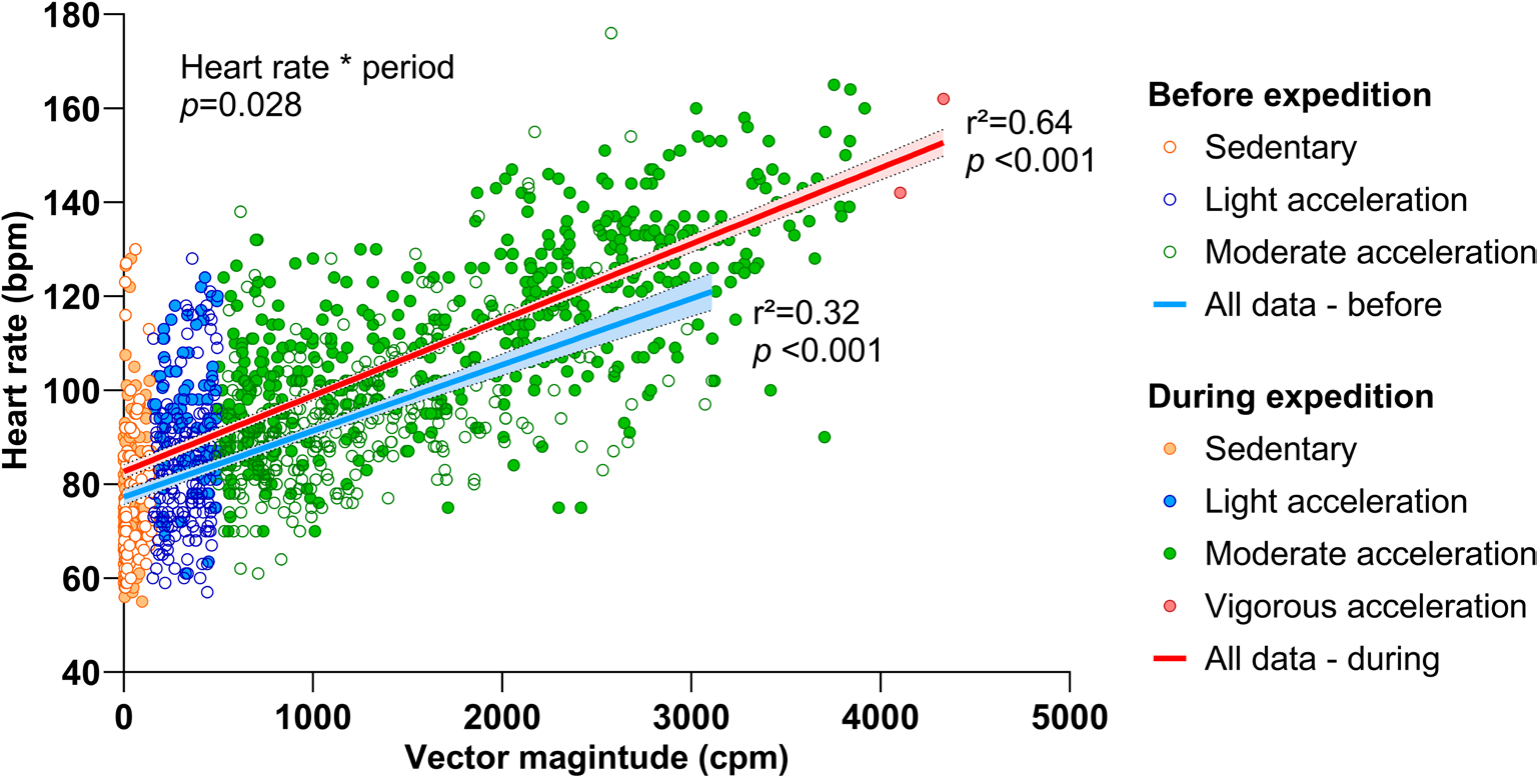
Association between heart rate and accelerometer‐derived body accelerations before (empty circle) and during (filled circle) the expedition. The least‐squares regression line before (blue) and during (red) the expedition is plotted, along with its 95% confidence interval, in a shaded area.

Compared to the pre‐expedition period (i.e., baseline), participants significantly increased time spent in light (+4.82 [SE 0.30] h/d, 95% CI 4.15–5.49) and moderate (+0.73 [0.32] h/d, 95% CI 0.01–1.45) accelerations‐related activities, while time spent in vigorous accelerations‐related activities remained unchanged (−0.08 [0.02] h/d, 95% CI −0.14 to −0.03). Time spent in sedentary behaviors decreased by 23.2 (2.0) % (95% CI −27.7 to −18.9). Heart rate was positively associated with accelerations both before (*r* ^2^ = 0.32; *p* < 0.001) and during the expedition (*r* ^2^ = 0.64; *p* < 0.001). However, the slope of this relationship was significantly steeper during the expedition compared to the baseline (*p* = 0.03) (Figure 2), suggesting higher energy expenditure for the same level of acceleration.

This shift in physical activity pattern resulted in exceptionally high levels of energy expenditure, with an average DLW‐derived TDEE of 18.66 (SD 1.72) MJ/d, primarily driven by a high AEE of 10.76 [1.34] MJ/d. PAL during the expedition was 3.1 (0.2). RMR after the expedition was 6.31 (SE 0.29) MJ/d, which was not significantly different from baseline values (+0.15 [SE 0.19] MJ/d; 95% CI −0.27 to 0.58).

Based on body composition changes, the average daily energy intake during the expedition was estimated at 13.00 (SD 5.59) MJ/d. This was insufficient to meet the high energy needs, resulting in a negative energy balance of −5.66 (5.09) MJ/d (EB vs. 0, *p* = 0.004). Participants had an average water turnover of 2.95 (0.26) L/d (Table 2).

### 3.4 Skin temperature and salivary cortisol during the expedition

**FIGURE 3:**
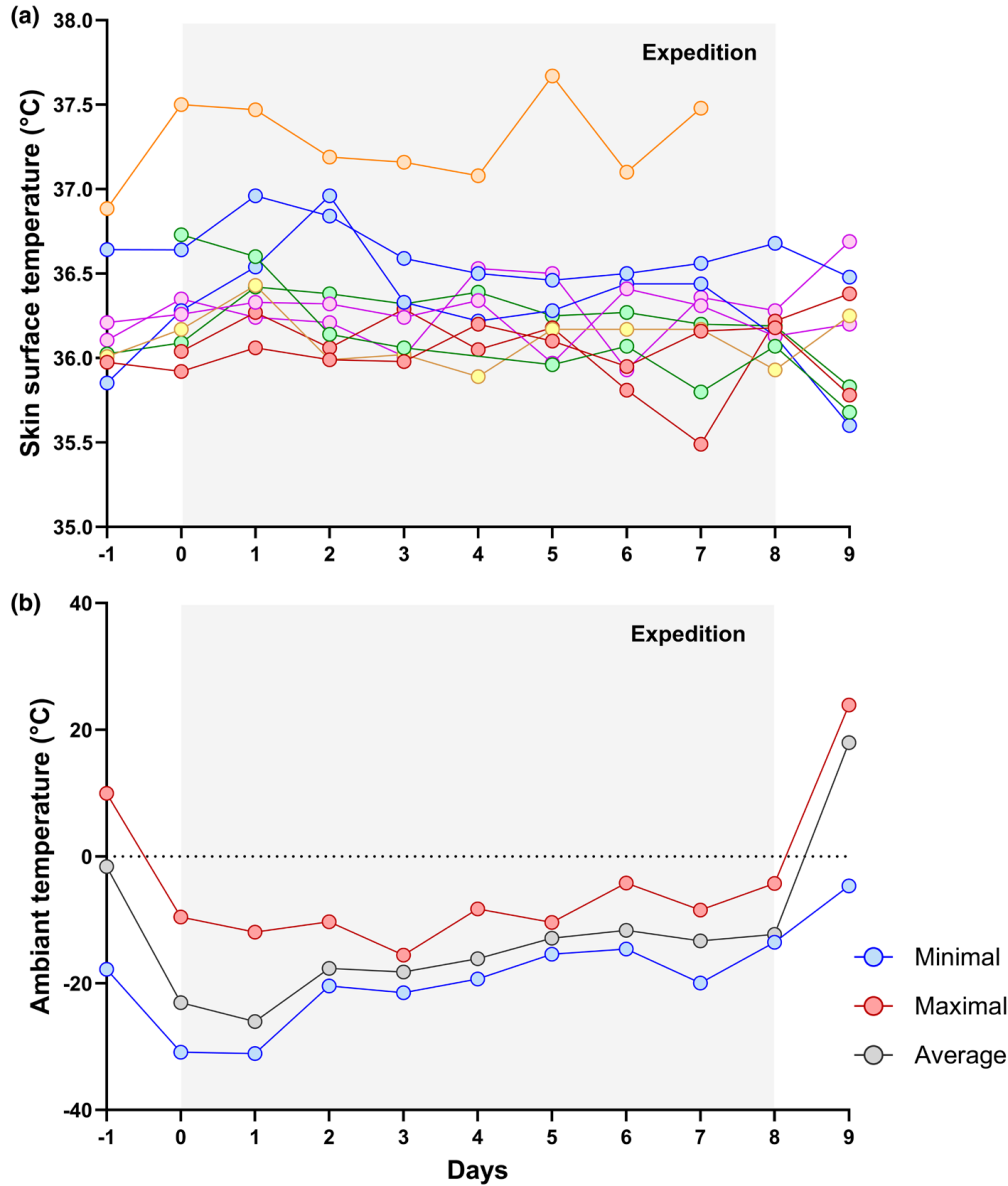
Evolution of individual skin surface temperature (a) and ambient temperature (b) before, during the expedition (shaded area) and after the expedition.

The mean daily ambient temperature during the expedition was −17.4°C, with minimum temperatures dropping as low as −31.1°C. Daily chest skin temperature showed a slight increase from 36.2 (SE 0.1)°C to 36.4 (0.1)°C (*p* = 0.03) (Figure 3).

**FIGURE 4:**
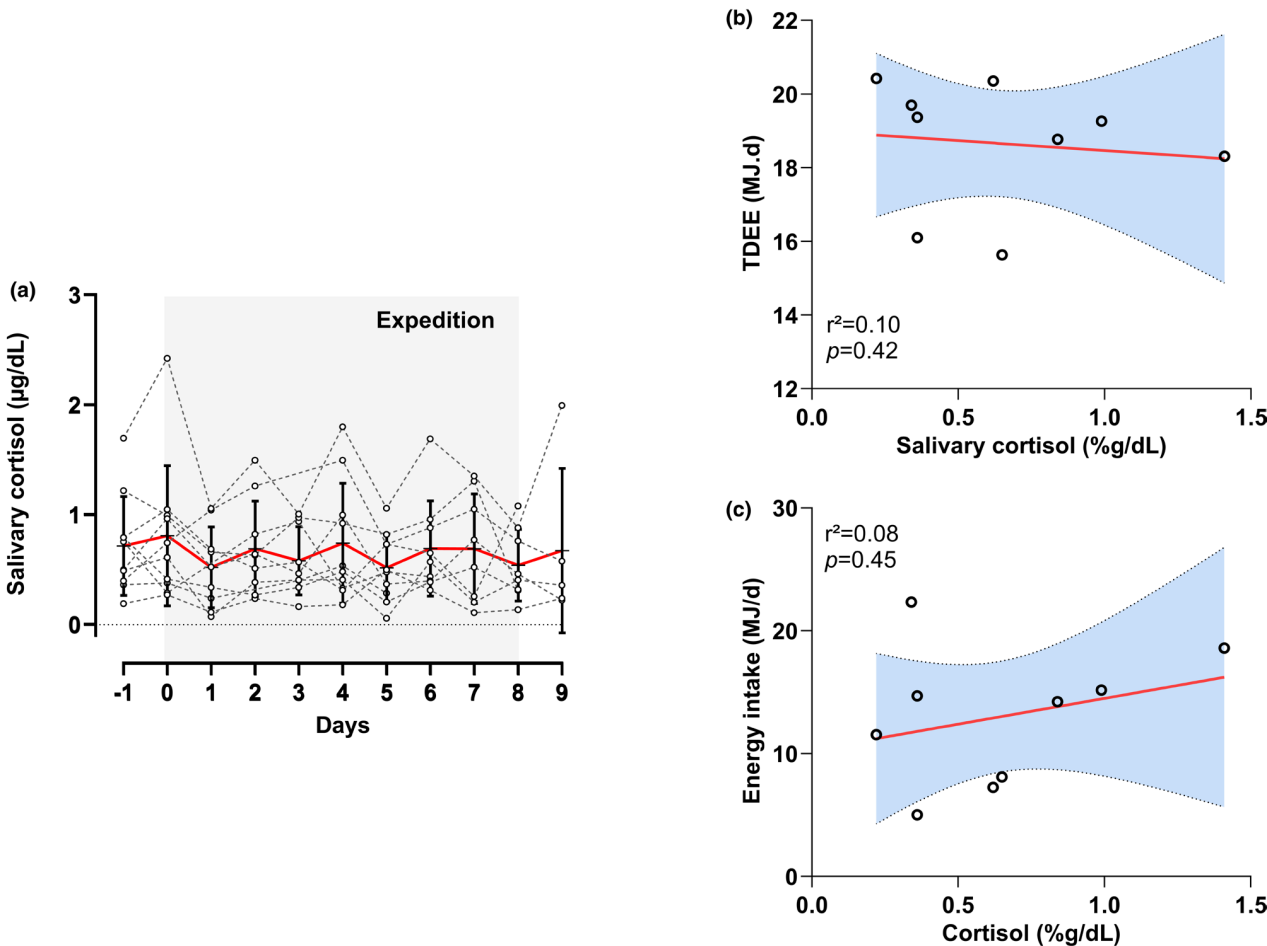
Evolution of salivary cortisol concentration before, during the expedition (shaded area) and after the expedition. Individual values are plotted with daily means and standard deviation (red line) (a). Associations between salivary cortisol and total daily energy expenditure (b) and energy intakes (c). The least squares regression line is plotted with the 95% confidence interval in a shaded area. TDEE, total daily energy expenditure.

Mean salivary cortisol concentration did not significantly change between the day before the expedition and the last day of the expedition (0.72 [SE 0.13] μg/dL vs. 0.63 [0.13] μg/dL, respectively; estimated difference + 0.09 [0.08] μg/dL, 95% CI −0.27 to 0.09). Nevertheless, substantial interindividual variability was observed, with baseline salivary concentration ranging from 0.19 to 1.69 μg/dL and post‐expedition concentrations ranging from 0.27 to 1.43 μg/dL (Figure 4). Cortisol levels were not associated with TDEE (*r* ^2^ = 0.10; *p* = 0.42) or energy intake (*r* ^2^ = 0.08; *p* = 0.45).

### 3.5 Sleep during the expedition

During the expedition, sleep duration increased by 0.50 h/night (SE 0.15; 95% CI 0.17–0.82), while the SRI score declined by 17.95 (2.85) points (95% CI −25.82 to 10.08) (Table 2).

## 4 DISCUSSION

The POWER study is the first to assess energy requirements in nonathlete, novice female explorers during a fully autonomous expedition to the North Pole. The findings revealed extremely high energy turnover rates, with a mean TDEE of 18.66 MJ/d, approximately 60% of which is attributable to AEE (10.76 MJ/d). Participants spent an average of 8.2 h per day engaged in light‐to‐moderate accelerations‐related activities, resulting in a PAL of 3.1. Body mass decreased by 2.42% compared to baseline values, primarily due to FM loss (−6.45% from initial values), indicating a significant energy deficit resulting from insufficient energy intake to meet these high energy demands.

TDEE measured in our participants is likely slightly underestimated, given logistical constraints during the first 24 h following the dose ingestion. These 24 h corresponded to a day in Longyearbyen to finalize the preparation of the material and rest for a few hours at the hotel before a 2‐h flight from Longyearbyen to Camp Barneo that took off around 1 am followed by a helicopter ride; participants remained seated during the whole trip. They started skiing immediately after the launch of the aircraft. Nevertheless, our findings are consistent with prior Arctic and Antarctic studies in men and women, where TDEE ranged from 22 to 27 MJ/d (Coker et al., 2025; Halsey & Stroud, 2012; Hattersley et al., 2020; Hoyt et al., 2001; Stroud et al., 1997). Variations across studies may be influenced by factors such as expedition duration, sled weight, ambient temperature, terrain, environmental conditions, and participants' characteristics such as body mass and composition. Interestingly, the TDEE observed in the POWER study aligns with those reported in extreme endurance sports, such as the Tour de France in cycling (Westerterp et al., 1986), the Race Across the USA in running (Thurber et al., 2019), or the Alaska Mountain Wilderness Ski Classic in ski trekking (Coker et al., 2025).

Since these high levels are reached and could be maintained over several days, the concern is more about the match between TDEE and EI, especially when the latter is limited (Charlot, 2021; Halsey & Stroud, 2012). The observed energy deficit (mean − 5.7 MJ/d) and fat mass loss (mean − 1.26 kg) suggest that participants were unable to consume enough food to meet their energy needs. This could be due to insufficient rations or the participants' inability to consume adequate food. Similar deficits have been documented during field military training, where EI was spontaneously reduced (Johnson et al., 2018; Jones et al., 1993). This volitional reduction in EI has been attributed to the willingness to spare food items in the case of prolonged event duration or the inability to refuel during the mission, as well as poor palatability, poor taste, and smell of dehydrated food rations (Ahmed et al., 2020). Previous cross‐sectional studies observed an incapacity to consume a sufficient amount of food when PAL values were above 2.2–2.5 in the general population (Melzer et al., 2005; Thurber et al., 2019). The present PAL of 3.1 in this novice explorer population may thus have contributed to the negative energy balance observed. Unfortunately, we could not precisely track food intake and appetite sensations during the expedition, which limited our understanding of these dynamics.

Stress may also play a role in reduced EI during polar expeditions (Rabasa & Dickson, 2016). While salivary cortisol concentration—used as a proxy of physiological stress level—did not significantly change during the expedition, the baseline samples were likely collected during a stressful preparation period, limiting their ability to reflect true pre‐expedition levels. In addition, no association was found between cortisol levels and TDEE or estimated EI, suggesting that other factors, such as environmental challenges of food quality, may have a greater impact.

Accelerometer data highlighted that light accelerations‐related physical activities were the predominant component of daily activity. In polar conditions, light accelerations‐derived physical activities cannot be directly equated to light‐intensity physical activities, that is, activities associated with low levels of oxygen consumption (1.5–2.99 MET) due to environmental and logistical factors. These include traversing rugged terrain, increased mechanical workload from polar suits (Teitlebaum & Goldman, 1972), and the added effort of pulling sleds weighing about 70 kg (Saibene et al., 1989). Such conditions associated with higher heart rates result in substantial energy expenditure despite slower movement speeds, as reflected in the high levels of AEE and TDEE (Leonard, 2003). Other physiological factors, such as dehydration, could also explain the steeper association between body acceleration and heart rate (Watso & Farquhar, 2019) during the expedition compared to baseline. Unfortunately, the effect of dehydration cannot be assessed as hydration status could not be objectively monitored during the trekking. Accelerometer‐derived data also highlighted longer median sleep duration during the expedition compared to baseline data. This may have been driven, in part, by sleep loss the night before the outset of the expedition. Conversely, sleep regularity was substantially lower during the expedition compared to the week prior, probably underscored by the constant daylight exposure.

Although the POWER study provides unique insights into energy balance regulation in nonathlete female explorers during a polar expedition—using gold standard methods in metabolic research, including DLW method, indirect calorimetry, and 3D‐acceleromtetry—certain limitations must be acknowledged.

The estimation of TDEE and its components relies upon several assumptions that may not be observed under the extreme environmental conditions of our experiment. First, the use of the DLW method assumes that participants are in energy balance, that is, TDEE equals energy intake. However, our participants were in a slight negative energy balance as indicated by the loss of fat mass. Because energy balance and fat balance are tightly linked (Schrauwen et al., 1998), this suggests a mismatch between FQ (used as a proxy for the 24‐h respiratory quotient under the assumption of energy balance) and the actual 24‐h respiratory quotient. Specifically, increased reliance on fat from adipose tissue lipolysis likely resulted in a lower respiratory quotient. We therefore likely underestimated TDEE and hence AEE during the expedition. Second, the cost of food processing may be greater than 10% during the polar expedition. Only a direct measurement of DIT in response to a standard meal under lab conditions and at the same temperatures they were exposed to during the expedition could have assessed this effect in these participants. Due to the significant challenges inherent in such a protocol, we had to assume that DIT was approximately 10% of TDEE on average, even under the extreme conditions of a polar environment and very high physical activity level. Third, estimating AEE by subtracting RMR from 90% of TEE is likely to suffer from cumulative methodological errors from both TDEE and RMR measurements. It has been previously discussed that this approach inherently introduces greater uncertainty compared to direct assessments, as it depends on multiple assumptions and compounds the variability of each individual measure (Gonzalez et al., 2023). In the absence of additional data on other components of energy expenditure, it may incorrectly attribute certain energy costs—such as those related to thermoregulation or fluctuations in RMR throughout the day—to physical activity (Gonzalez et al., 2023). Finally, the coefficient used for estimating FFM hydration status (0.73) may also be in accurate in case of dehydration of the volunteers following the expedition. This being said, as body composition was measured 16–24 h after their return to Longyearbyen, it is very likely that they rehydrated.

The pre‐expedition period likely did not represent habitual baseline conditions due to the physical and mental demands of preparation. Habitual stress levels, daily physical activity, and TDEE were not measured, and energy intake during the expedition was only estimated. Furthermore, we did not measure other physiological factors, such as shivering and non‐shivering thermogenesis, which are critical for understanding energy expenditure in extreme cold (Yurkevicius et al., 2022), or the cost of locomotion and mechanical efficiency. Although skin temperature was recorded at the chest, it serves as a poor proxy for thermoregulation. These limitations underscore the need for improved technologies and methodologies to comprehensively assess energy expenditure and intake in extreme environments. Such advancements could deepen our understanding of how physical activity, thermoregulation, stress, and disturbed sleep affect energy balance regulation during polar expeditions.

## AUTHOR CONTRIBUTIONS

AB, JD, SG, CS, and SB conceived and designed research. AB, JD, and SG performed experiments. PB, AZ, IC, CS, and AB analyzed data. PB, AZ, IC, CS, SB, and AB interpreted results of experiments. PB prepared figures and drafted manuscript. PB, CS, AB, and SB edited and revised manuscript. All the authors approved final version of manuscript.

## FUNDING INFORMATION

French Center for Scientific Research (CNRS) Mission for transversal and interdisciplinary initiatives (MITI) and the Doctoral School of Lice Science (ED 414) of the University of Strasbourg, France.

## CONFLICT OF INTEREST STATEMENT

The authors declare that they have no potential conflicts of interest that might be relevant to the contents of this manuscript.

## ETHICS STATEMENT

The study was approved by the Colorado Multiple Institutional Review Board (COMIRB) under protocol number 17‐2144. Written informed consent was obtained from all participants in accordance with the principles of the Declaration of Helsinki.

## Supporting information


Table S1.

## Data Availability

Data are accessible upon reasonable request to the authors.
