# Supplementary material for: Energy balance in women during polar trekking—The POWER study
Source: Physiol Rep. 2025 Jun 30;13(13):e70443. doi: 10.14814/phy2.70443 (PMC12208795; doi:10.14814/phy2.70443)
Supplement: Supplementary file 1 — Table S1. [file PHY2-13-e70443-s001.docx]

RESEARCH ARTICLE

Supplemental file

Energy balance in women during polar trekking – the POWER study

Pierre Bourdier ^1,2^, Jessica Devitt ^3^, Susan Gallon ^4^, Alexandre Zahariev ^1^, Isabelle Chery ^1^, Jacob R. Guzzetti ^5^, Stéphane Blanc ^1^, Chantal Simon ^6,7^, and Audrey Bergouignan ^1,8*^

^1^ Université de Strasbourg, CNRS, IPHC UMR7178, F-67000 Strasbourg, France

^2^ Université de Haute-Alsace, IRIMAS UR7499, F-68100 Mulhouse, France

^3^ Department of Family Medicine, University of Colorado, Anschutz Medical Campus, Aurora, CO, USA

^4^ Network of Marine Protected Areas in the Mediterranean (MedPAN), Marseille, France

^5^ Behaviour-Brain-Body Research Centre, University of South Australia, Adelaide, Australia

^6^ CarMen Laboratory, INSERM 1060, INRAE 1397, University of Lyon, Oullins, France

^7^ Human Nutrition Research Centre of Rhône-Alpes, Hospices Civils de Lyon, Lyon, France

^8^ Division of Endocrinology, Metabolism and Diabetes, Anschutz Health & Wellness Center, University of Colorado, Anschutz Medical Campus, Aurora, CO, USA

Correspondence: *Audrey Bergouignan (audrey.bergouignan@iphc.cnrs.fr); Pierre Bourdier (pierre.bourdier@uha.fr)*

**Table S1.** Macronutrient composition and calories of food intake during the expedition for prepacked meals (a) and additional snacks (b)

| **Pre-packed**  **meals (a)** | **Macaroni Cheese** | | **Salmon and Brocoli Pasta** | | **Chicken Fried Rice** | | **Beef and Potato Stew** | | **Chicken Tikka with Rice** | | **Custard Apple Crunch** | | **Rice Pudding with Strawberry** | | **Pasta Bolognaise** | | **Morning oats with rasberry** | | **Chicken fajita with rice** | |
| --- | --- | --- | --- | --- | --- | --- | --- | --- | --- | --- | --- | --- | --- | --- | --- | --- | --- | --- | --- | --- |
|  | Per 100g | Per pack | Per 100g | Per pack | Per 100g | Per pack | Per 100g | Per pack | Per 100g | Per pack | Per 100g | Per pack | Per 100g | Per pack | Per 100g | Per pack | Per 100g | For 10 packs* | Per 100g | Per pack |
| **EI (kJ)** | 2246 | 2516 | 2275 | 2661 | 2135 | 2583 | 2199 | 2595 | 2280 | 2873 | 1873 | 2153 | 1698 | 1974 | 2280 | 3100 | 2072 | 18850 | 2090 | 2675 |
| **EI (kcal)** | 539 | 603 | 545 | 638 | 510 | 617 | 527 | 622 | 546 | 688 | 447 | 514 | 404 | 469 | 545 | 741 | 495 | 4505 | 500 | 639 |
| **Fat (g)** | 34 | 38 | 32 | 37 | 23 | 28 | 31 | 36 | 30 | 38 | 22 | 25 | 13 | 16 | 28 | 38 | 19 | 18 | 22 | 29 |
| **Carbohydrate (g)** | 38 | 43 | 48 | 56 | 50 | 61 | 39 | 46 | 51 | 64 | 54 | 61 | 64 | 75 | 51 | 69 | 66 | 60 | 52 | 67 |
| **Protein (g)** | 20 | 22 | 16 | 18 | 21 | 26 | 20 | 24 | 16 | 20 | 7 | 9 | 6 | 6 | 19 | 26 | 11 | 10 | 19 | 24 |
| **Fat (%)** | 36.8 | | 33.3 | | 24.6 | | 34.3 | | 31.0 | | 26.6 | | 16.1 | | 28.4 | | 2.0 | | 24.0 | |
| **Carbohydrate (%)** | 41.5 | | 50.3 | | 52.9 | | 43.5 | | 52.8 | | 64.4 | | 77.3 | | 52.1 | | 6.9 | | 55.8 | |
| **Protein (%)** | 21.8 | | 16.4 | | 22.5 | | 22.3 | | 16.2 | | 8.9 | | 6.6 | | 19.6 | | 1.1 | | 20.2 | |

| **Additional**  **snacks (b)**** | **Chocolate + caramel bar** | **Chocolate bar** | **Candy 1** | **Candy 2** | **Chocolate + milk biscuit** | **Sport bar** | **Raspberry** | **Almond** |
| --- | --- | --- | --- | --- | --- | --- | --- | --- |
| **EI (kJ)** | 1021 | 933 | 1230 | 958 | 9012 | 799 | 247 | 2497 |
| **EI (kcal)** | 244 | 223 | 294 | 229 | 2154 | 191 | 59 | 597 |
| **Fat (g)** | 12 | 7 | 11 | 10 | 17 | 98 | 1 | 54 |
| **Carbohydrate (g)** | 32 | 25 | 28 | 32 | 17 | 215 | 12 | 5 |
| **Protein (g)** | 2 | 7 | 3 | 19 | 2 | 103 | 1 | 22 |
| **Fat (%)** | 26.1 | 26.2 | 16.4 | 47.2 | 23.6 | 17.9 | 5.0 | 66.3 |
| **Carbohydrate (%)** | 69.6 | 66.7 | 52.5 | 47.2 | 51.7 | 64.1 | 86.3 | 6.6 |
| **Protein (%)** | 4.3 | 7.1 | 31.1 | 5.6 | 24.8 | 17.9 | 8.6 | 27.1 |

EI= energy intake

* One morning oats with raspberry per morning

** Supplementary snacks were made available in bulk for shared team use (i.e.; not individual portions).
